# Adaptive immune receptor genotyping using the *corecount* program

**DOI:** 10.3389/fimmu.2023.1125884

**Published:** 2023-04-11

**Authors:** Sanjana Narang, Mateusz Kaduk, Mark Chernyshev, Gunilla B. Karlsson Hedestam, Martin M. Corcoran

**Affiliations:** Department of Microbiology, Tumor and Cell Biology, Karolinska Institutet, Stockholm, Sweden

**Keywords:** IGH, VDJ germline genes, immune repertoires, inference, genotyping

## Abstract

We present a new Rep-Seq analysis tool called *corecount*, for analyzing genotypic variation in immunoglobulin (IG) and T cell receptor (TCR) genes. *corecount* is highly efficient at identifying V alleles, including those that are infrequently used in expressed repertoires and those that contain 3’ end variation that are otherwise refractory to reliable identification during germline inference from expressed libraries. Furthermore, *corecount* facilitates accurate D and J gene genotyping. The output is highly reproducible and facilitates the comparison of genotypes from multiple individuals, such as those from clinical cohorts. Here, we applied *corecount* to the genotypic analysis of IgM libraries from 16 individuals. To demonstrate the accuracy of *corecount*, we Sanger sequenced all the heavy chain IG alleles (65 IGHV, 27 IGHD and 7 IGHJ) from one individual from whom we also produced two independent IgM Rep-seq datasets. Genomic analysis revealed that 5 known IGHV and 2 IGHJ sequences are truncated in current reference databases. This dataset of genomically validated alleles and IgM libraries from the same individual provides a useful resource for benchmarking other bioinformatic programs that involve V, D and J assignments and germline inference, and may facilitate the development of AIRR-Seq analysis tools that can take benefit from the availability of more comprehensive reference databases.

## Introduction

The ability of the adaptive immune system to respond efficiently to pathogens is predicated on its capacity to generate vast numbers of individual receptors with different specificities through a recombination process that joins variable (V), diversity (D) and junction (J) segments in the case of IGH, TRB and TRD genes and V and J genes, in the case of IGK, IGL, TRA and TRG genes (1). The recombination process generates the complementarity determining region 3 (CDR3), which is characteristic for each B or T cell receptor and is a major specificity determinant for most B and T cells. In addition to the stochastic generation of CDR3 diversity during the recombination process, the adaptive immune receptor gene segments that comprise the building blocks of the receptors are themselves a source of variation, both at the individual and the population level (2). This genetic diversity is of two main types. The first, structural variation (SV), involves deletion or duplication of genomic segments that contain one of more V, D, J, or constant region genes (3). The second, allelic diversity, comprises nucleotide level variation, several of which are known to facilitate antibody binding to pathogen associated epitopes (4–6). A major issue that affects the analysis of immunoglobulin gene variants in different diseases involves the complex nature of the IG (immunoglobulin) loci (7–9). The heavy and light chain loci have evolved over millions of years through a process of gene duplication with the result that each loci contains many unique genes and chromosomal segments containing multiple genes that differ only slightly from other genes in the loci (10, 11). In addition, over time many of these duplicated genes have become non-functional pseudogenes that nevertheless share significant sequence identity with evolutionary related functional genes. The combination of these factors results in genomic regions that are refractory to short read sequence analysis (12).

This structural and allelic diversity results in collections of expressed functional germline sequences that differ between individuals. A collection of allelic variants derived from the union of unique V, D and J alleles found in many individuals that can then be used as a reference set is generally termed a database. The creation of comprehensive germline databases is a topic of intense interest, with multiple independent approaches attempting to address this issue. While genomic-based methods are increasingly being utilized, expression-based germline inference is a well-established process that has been used to identify multiple novel alleles in human and other species and has the advantage that the identified alleles are functional as they are found in expressed rearrangements in the repertoire (13–15). In general, germline inference from expressed sequences involves the assignment of sequences to the closest member of an initial or ‘starting ‘database, followed by the computational identification of candidate germline sequences that differ in a consistent way from one or more of the starting database sequences. In the case of germline IGH sequences, the availability of IgM libraries that contain a substantial proportion of naïve, unmutated sequences, enables the inference of novel alleles that differ by as little as one nucleotide from reference alleles in the starting database (13, 15, 16).

While germline inference is a valuable tool for the identification of novel variants present in an individual, it can be time-costly in the case of large datasets that may require frequent re-analysis using updated or modified starting databases. In addition, ongoing work of multiple groups in identifying novel allelic variants in collections comprised of samples from many human population groups will inevitably mean that most common or even rarer variants present in most human populations will be identified within the next few years (17). In this scenario the individualized genotype approach can shift emphasis from inference of novel alleles to the assessment of genotype using a well-defined and comprehensive database. The current study examines one approach to utilizing more comprehensive databases as a means of rapidly genotyping cases from expressed AIRR (Adaptive Immune Receptor Repertoire) libraries. We performed this analysis based on detailed knowledge of the genomic sequences encoding all expressed IGHV, IGHD and IGHJ alleles in a single gene and allele rich case, obtained by targeted genomic PCR, cloning and Sanger sequencing to ensure high fidelity genotype validation. The validation process also enabled the correction of multiple erroneously truncated alleles from the reference database, and allowed the creation of an end-corrected, full-length allele reference database.

Based on this foundation, we developed a novel computational approach termed *corecount* that allows high-confidence identification of germline alleles from IgM libraries, thereby producing a genotype of functional V, D and J alleles for each individual. We confirmed the accuracy of the process by applying *corecount* to two IgM libraries, generated by two different primer sets, from the same individual as was used for the targeted genomic PCR, thereby providing three independent datasets for this case. We show that *corecount* can be used for IG V, D and J genotyping from IgM libraries that have been pre-filtered by IgDiscover, and that even genes that are infrequently used in the IgM repertoire can be identified accurately using this approach.

## Results

### Overall work scheme and inferred haplotype analysis of D19

**Figure 1:**
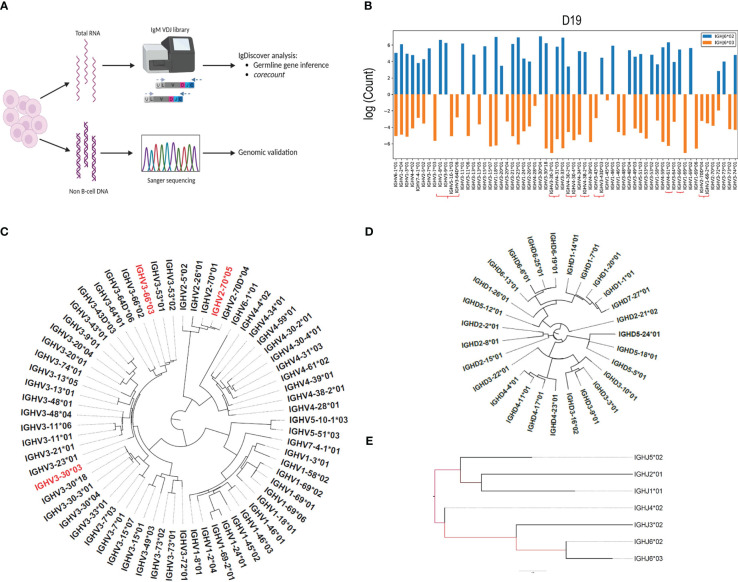
IgM library preparation and inferred genotype. **(A)** Peripheral blood samples separated into polymorphonuclear and mononuclear cell fractions, with DNA and RNA extracted from each fraction respectively. IgM libraries were prepared from the RNA fraction, sequenced using the Illumina MiSeq system, and analyzed for the presence of novel alleles using the IgDiscover program. Genotyping was subsequently performed using the *corecount* module. DNA isolated from the polymorphonuclear fraction was used as template for genomic PCR of individual V, D and J alleles and subsequent Sanger validation. **(B)** Inferred haplotype analysis of case D19. IgDiscover plotallele output using IGHJ6*02 and IGHJ6*03 as haplotyping anchors. Genes associated with common structural alterations are marked with the red brackets. Dendrograms of **(C)**, Sanger validated IGHV alleles, with non-expressed alleles denoted in red, as revealed by their lack of expression in two independent IgM libraries, **(D)**, IGHD alleles, and **(E)**, IGHJ alleles from case D19.

We combined the use of IgM library preparation, NGS (Next generation sequencing) and IGH germline gene inference using IgDiscover to identify a case with a high IGH gene content (**Figures 1A–E**). We used IgM Rep-seq data from 14 individuals for whom we had previously defined their germline IGH V, D and J genotypes with IgDiscover (18), and two additional cases, D19 and D46. We subsequently focused on D19, who was found to be heterozygous for IGHJ6*02/*03 and contained a total of 62 expressed IGHV alleles. We identified which of the IGHV alleles were present on each chromosome through inferred haplotyping using the IgDiscover plotallele module (**Figure 1B**). D19 showed heterozygosity for the common IGHV structural variant containing IGHV1-8 and IGHV3-9 on one haplotype and IGHV5-10-1 and IGHV3-64D on the other. Additionally, D19 contained alleles of IGHV3-30-3, IGHV4-31, IGHV4-30-2, IGHV4-30-4, IGHV3-43D, IGHV4-38-2, IGHV4-61, IGHV3-66, IGHV1-69-2 and IGHV2-70D, which are known to be absent from many haplotypes (19). Due to the high number of expressed genes and alleles we chose this case as suitable for demonstrating the *corecount* approach. Furthermore, we recognized that the availability of genomic IGH gene sequences from the same case would allow for a detailed validation of the inference outputs and would provide a useful resource for the AIRR community to benchmark subsequent tools for germline gene inference and personalized VDJ genotyping.

### Genomic validation of D19 germline alleles

**Figure 2:**
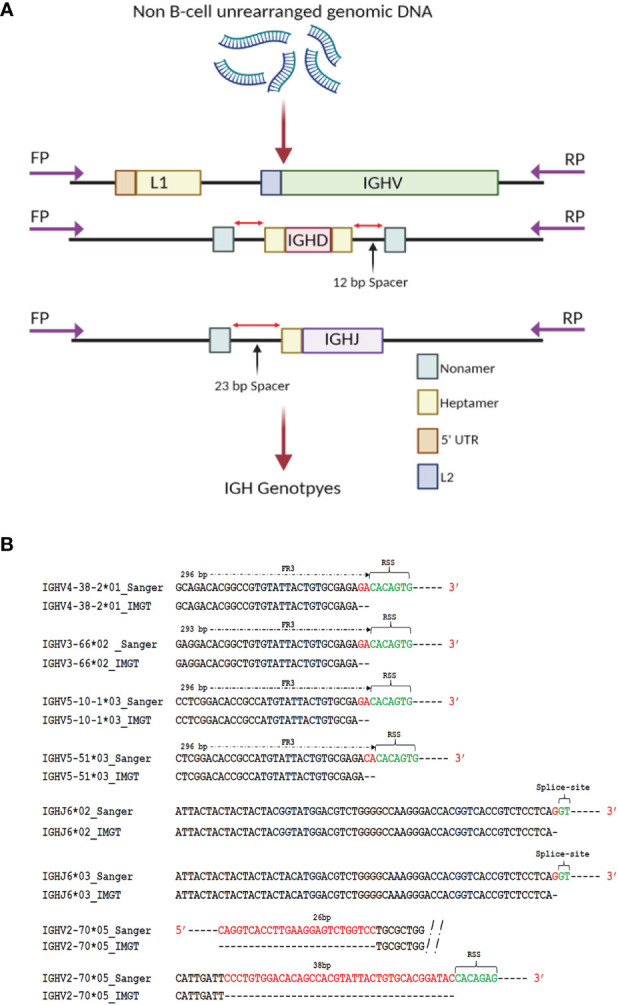
Genomic validation. **(A)** DNA extracted from polymorphonuclear cells from case D19 was used as template for individual PCR amplification of all IGHV, IGHD and IGHJ genes. Primers were located upstream of the leader coding sequence, and downstream of the RSS sequence in L2 V exon. Primers targeting the D genes were located upstream and downstream of the 5’ and 3’ D gene RSS segments respectively. Primers targeting the J gene were located upstream of the RSS segment and downstream of the J gene splice site segment. **(B)** Sequence discrepancies of known reference alleles identified through Sanger validation. Full length extension of truncated alleles IGHV3-66*02, IGHV4-38-2*01, IGHV2-70*05, IGHV5-10-1*03, IGHV5-51*03, IGHJ6*02 and IGHJ6*03 was enabled by the identification of the positions of framework 1, RSS heptamer sequence or splice sites within the genomic sequence encompassing the truncated allelic sequences.

To generate genomic IGH gene sequences from this case, we used targeted PCR followed by cloning and Sanger sequencing of all 62 expressed IGHV alleles, 27 IGHD alleles and 7 IGHJ alleles (**Figure 2A** and **Supplemental Table 1**). Sequence analysis of seven known reference germline alleles, IGHV5-10-1*03, IGHV5-51*03, IGHV4-38-2*01, IGHV3-66*02, IGHV2-70*05, IGHJ6*02 and IGHJ6*03 revealed that each were longer compared to the IMGT (International ImMunoGeneTics) reference sequences (**Figure 2B**). The additional nucleotides (nts) in four IGHV sequences, IGHV3-66*02, IGHV4-38-2*01, IGHV5-10-1*03 and IGHV5-51*03, were present just before the RSS heptamer, indicating short truncations in the reference database sequences, with all four of the reference IGHV alleles missing 2 nts. An additional V allele, IGHV1-69*02 was truncated by 2 nts compared to the allele in the reference database initially downloaded in May 2020. This allele has, however, been extended recently in the IMGT database to add two additional nts and the updated IGHV1-69*02 sequence is consistent with that found in our Sanger validated sequence. During the Sanger validation process we identified three additional alleles, IGHV2-70*05, IGHV3-66*03 and IGHV3-30*03 that were present at the genomic level but were not present in the IgDiscover output for D19 (shown in red in **Figure 1C**), in either the leader primer set library or the upstream primer set library. For the non-expressed Sanger validated sequence that showed 100% identity to the reference allele IGHV2-70*05, our sequence contained an extra 26 nts in the 5’ end of the V and 38 nts at the 3’ end of the V, and in contrast to the reference allele, is of identical length to other full-length alleles of IGHV2-70. Two additional known alleles, IGHV3-30*03 and IGHV3-66*03, were also not present in the IgDiscover output but identified in the genomic Sanger sequence data (**Supplemental Table 1**). The final two truncated alleles, IGHJ6*02 and IGHJ6*03, both contained an additional G nucleotide immediately prior to the GT splice donor site of this gene, indicating a truncation of 1 nt in the IGHJ6*02 and IGHJ6*03 reference sequences (**Figure 2B** and **Supplemental Table 1**).

### Identification of V, D and J core sequences

**Figure 3:**
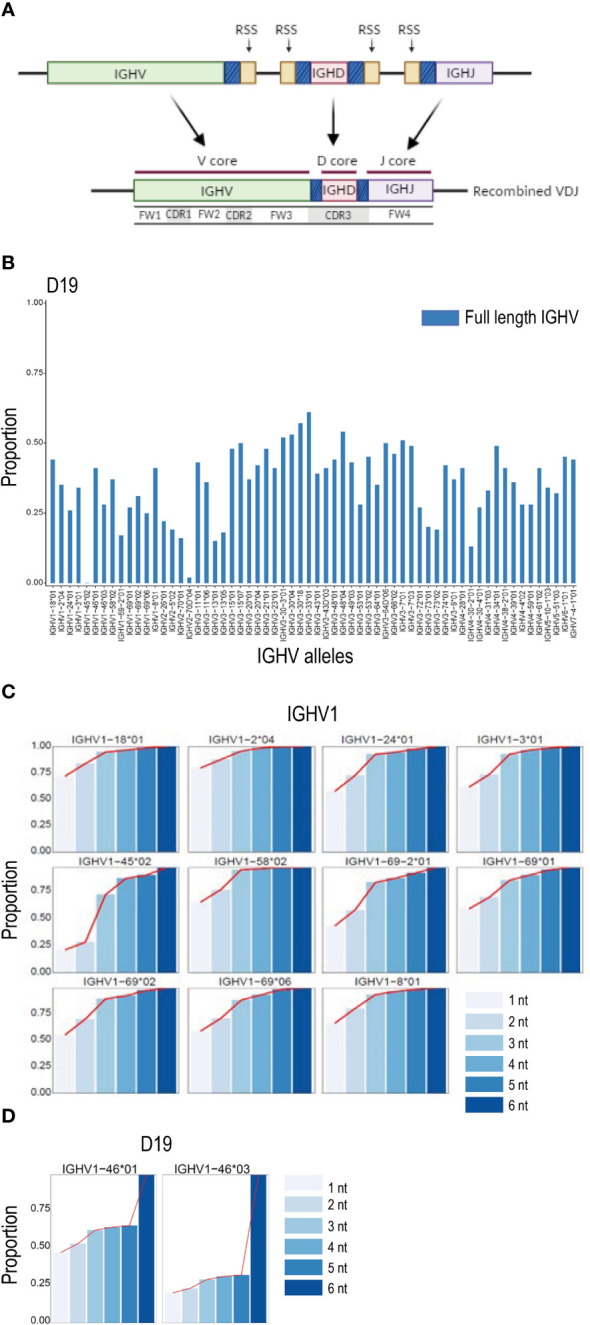
VDJ associated IGHV truncation. **(A)** Schematic of VDJ recombination. Genomically distinct V, D and J alleles are recombined to produce a unique VDJ ‘exon’. The recombination process in most cases causes loss or replacement of several 3’ located nucleotides of the V gene, 5’ nucleotides of J genes and both 5’ and 3’ nucleotides of D genes, thereby enabling the generation of high levels of CDR3 coding diversity. The sections of the V, D and J genes subject to high rates of recombination associated change are denoted with a shaded blue color in the figure. The gene segments least affected by the recombination process are termed here the V, D and J core sequences. **(B)** Effect of recombination based 3’ nucleotide alteration on unmutated allele counts in D19 using NGS analysis of the IgM library. The proportion of counts for V alleles containing the entire V sequence are shown in blue. **(C)** The proportion of counts of a series of single nucleotide 3’ deletions are shown compared to the counts of a 6 nt truncation at the 3’ end of the V sequence for IGHV1 alleles in case D19. In all cases a plateau of counts is achieved at the -4 or -5 nt point. **(D)** Proportion of unmutated allele sequence of the gene IGHV1-46 containing nucleotide differences close to the IGHV 3’ end increases rapidly when the allelic search string is decreased by 5 nucleotides at the3 ’ end due to the presence of the single G/T snp that distinguishes IGHV1-46*01 from IGHV1-46*03 in case D19.

The IGH VDJ recombination process results in nucleotide additions or trimming of germline sequences within the regions of V, D and J genes in the close vicinity of the RSS site (**Figure 3A**). We investigated the effect of the recombination-mediated alteration of the IGHV 3’ region on the ability to detect full length non-mutated allelic sequences in IgM libraries by comparing the proportion of counts of V sequences identical to the full-length V allele and, alternatively, the V sequence truncated to the conserved cysteine position at the CDR3 border, a length of approximately eight nucleotides. For all IGHV alleles in D19, the truncated sequences resulted in counts that were at least two to four times higher than the counts for the full-length allelic sequences (**Figure 3B**). Similar results were obtained when analyzing another individual, D46 (**Supplemental Figure 1A**). To investigate further, we performed iterative single nucleotide truncation counts from the 3’ end for each V allele. This allowed us to determine whether the sequence counts increased linearly up to a certain point as the V sequence was 3’ truncated. Alternatively, this process would identify the region primarily affected by recombination and delineate the section primarily unaffected, termed here the ‘core’ sequence. The results revealed that trimming the final two nucleotides of the reference V sequence resulted in a proportional increase in counts for most alleles, and further increases in unmutated V allele counts was found for each additional single nucleotide reduction in the V search sequence (**Figure 3C** and **Supplemental Figure 1B**). The results also demonstrated that a plateau was reached for all V sequences at a truncation of approximately 4 nucleotides. In the case of heterozygous alleles that differ solely at positions close to the 3’ end of a gene, truncation past the variant nucleotide that distinguishes the two alleles resulted in a sudden increase in the combined allele count since the two alleles could no longer be distinguished from each other (**Figure 3D** and **Supplemental Figure 1C**). Analysis of the effect of reference sequence truncation on IGHJ and IGHD count was also investigated, revealing similar increases in count levels when the regions affected by recombination were excluded (**Supplemental Figures 2A, B**).

### The principle of *corecount* genotype analysis

**Figure 4:**
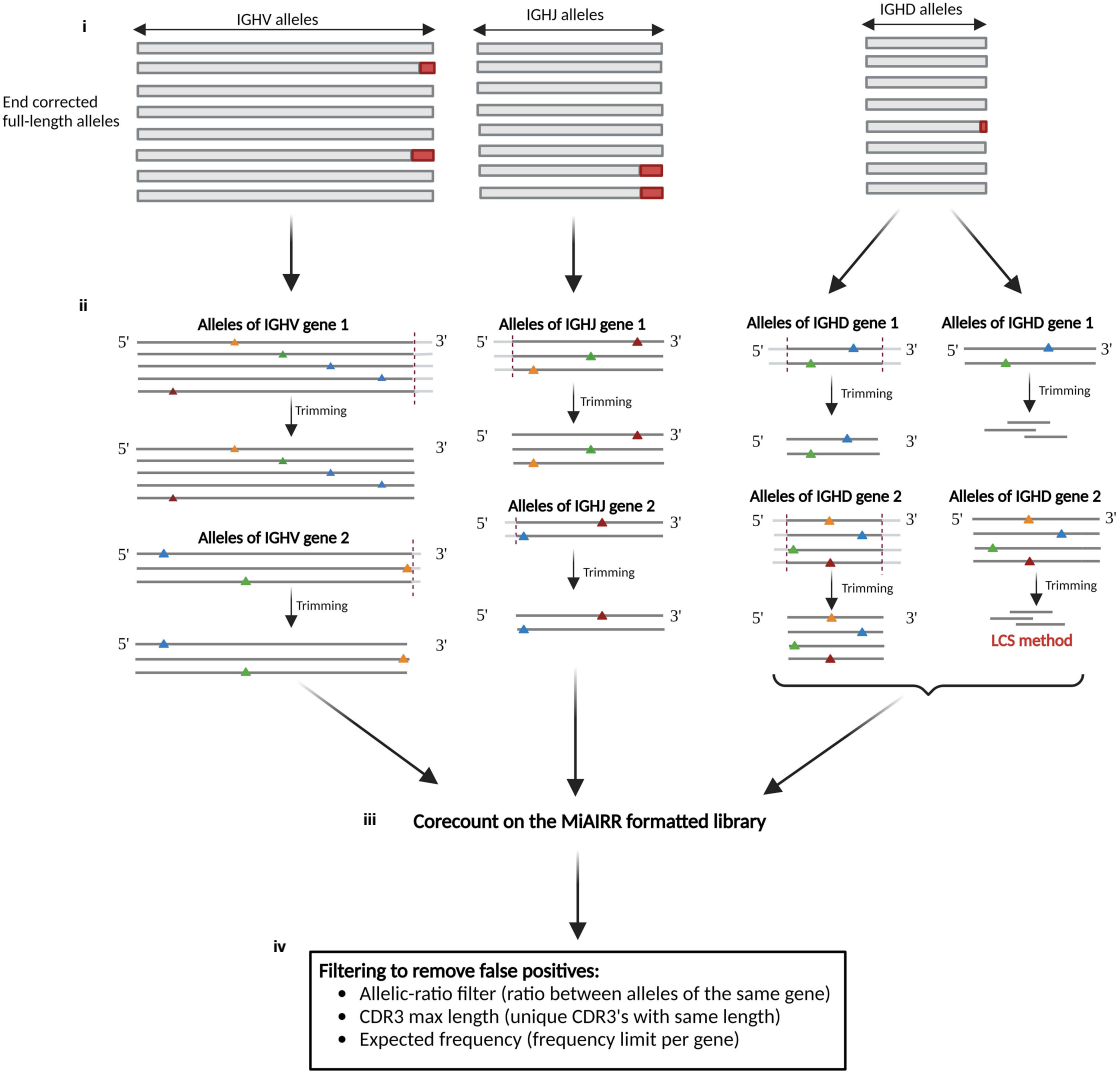
Schematic of *corecount* process. **(i)** The starting point for *corecount* analysis is the availability of a validated comprehensive database that contains end corrected allelic sequences. **(ii)** Database processing by *corecount*. The program processes allelic variants on a per gene basis, truncating the database sequences at the appropriate regions affected by VDJ recombination associated variation, trimming a default or user defined number of nucleotides from each sequence in the recombination associated regions, to leave a ‘core’ sequence specific for each allele. In the case of V genes where one or more allelic variant that distinguishes this allele from another is present within the last three nucleotides of the 3’ end of the gene, *corecount* truncates the sequence of all alleles of this gene only as far as that variant nucleotide. In each case, within a single gene all *corecount* processed alleles will be truncated by the same amount, facilitating a direct comparison of the counts for each expressed allele. The *corecount* database truncation occurs at the 3’ end of V sequences, 5’ end of J sequences, and both 5’ and 3’ end of D sequences. For D gene analysis, *corecount* additionally includes an alternative procedure that involves identifying the longest common substring (LCS) of allele specific sequences within the D gene sequences that are used as alternative core sequence search strings. **(iii)** *corecount* analysis of MiAIRR formatted NGS library. The *corecount* program identifies sequence matches to the database sequences within the library and passes the raw sequence counts to the germline filter step. **(iv)** Filtering of raw *corecount* output to produce the final expressed genotype. This filter step that can be user defined, includes an allelic ratio that compares counts of all alleles of each individual gene, a minimum count requirement, an expected frequency based on gene and allele frequency from multiple independent libraries, and two CDR3 diversity filters that detect and remove false positive expanded clones based on either biased CDR3 length or sequence diversity amongst the set of CDR3s associated with each germline sequence. The output of the program is a genotype of the appropriate gene type (V, D or J).

The *corecount* process (**Figure 4**) derives allele-specific search strings from the set of V, D or J sequences contained within a reference database of which each allelic sequence is ensured to be full length, hence a reference database containing truncated alleles is unsuitable as a starting database for *corecount*. The processed database sequences are designed to recognize the part of each allele that is, in most instances, unaffected by VDJ recombination, termed the ‘core’ sequences. The core sequences are used to search within a MiAIRR format filtered library table, in this case the output of an IgDiscover analysis, to utilize information about unique molecular identifiers (UMI) counts and CDR3 diversity that the program requires for germline filtering. The core processing is different for V, D and J genes. It begins with ensuring the database is suitable for *corecount* analysis (**Figure 4i**). For V germline genes, the program first tests whether an allele-specific variant nucleotide is present within 6 nts of the 3’ end of the reference alleles of each gene. If no such variant nucleotides are located within the segment, then all V reference alleles are shortened by a default or user-defined length (**Figure 4ii**). This database truncation ensures that *corecount* can take advantage of the increase in counts of unmutated sequences found outside the V recombination region. If, however, a variant nucleotide is identified within a segment that distinguishes one allele from other alleles of a specific gene, all alleles of that gene are shortened to exactly the length that preserves the sole variant nucleotide, i.e., the reference alleles of that gene in the *corecount* database may instead be truncated by two, one or even zero nts in the case of a gene where there is an allele with a single variant position at the final nucleotide.

In contrast to 3’ trimmed IGHV core sequences, IGHJ sequences for *corecount* analysis are trimmed at the 5’ end of the J sequence as this is the section of the J gene affected by recombination (**Figure 4ii**, middle), and IGHD alleles are truncated at both the 5’ and the 3’ ends (**Figure 4ii**, right). Genotypic analysis of IGHD and IGHJ genes from IgM libraries is enabled within the *corecoun*t module with user specified settings. For IGHD genotype analysis, several additional factors must be considered. First, database processing must ensure that the allelic core sequences are gene specific and of sufficient length to distinguish between IGHD genes of similar sequence, for example genes of the IGHD1 gene family. When SHM or instrument associated single nucleotide errors occur, due the size of D genes, the chance of this resulting in erroneous allele counts is higher compared to the same situation with IGHV and IGHJ sequences. The *corecount* module therefore compensates for this by utilizing a higher default allelic ratio setting for D gene genotyping. The accuracy of D allele genotyping when utilizing the default 0.2 cutoff settings was determined using the Sanger validated case D19, with the *corecount* results showing 100% concordance between the expressed and genomic genotypes (**Supplemental Table 2**).

**Figure 5:**
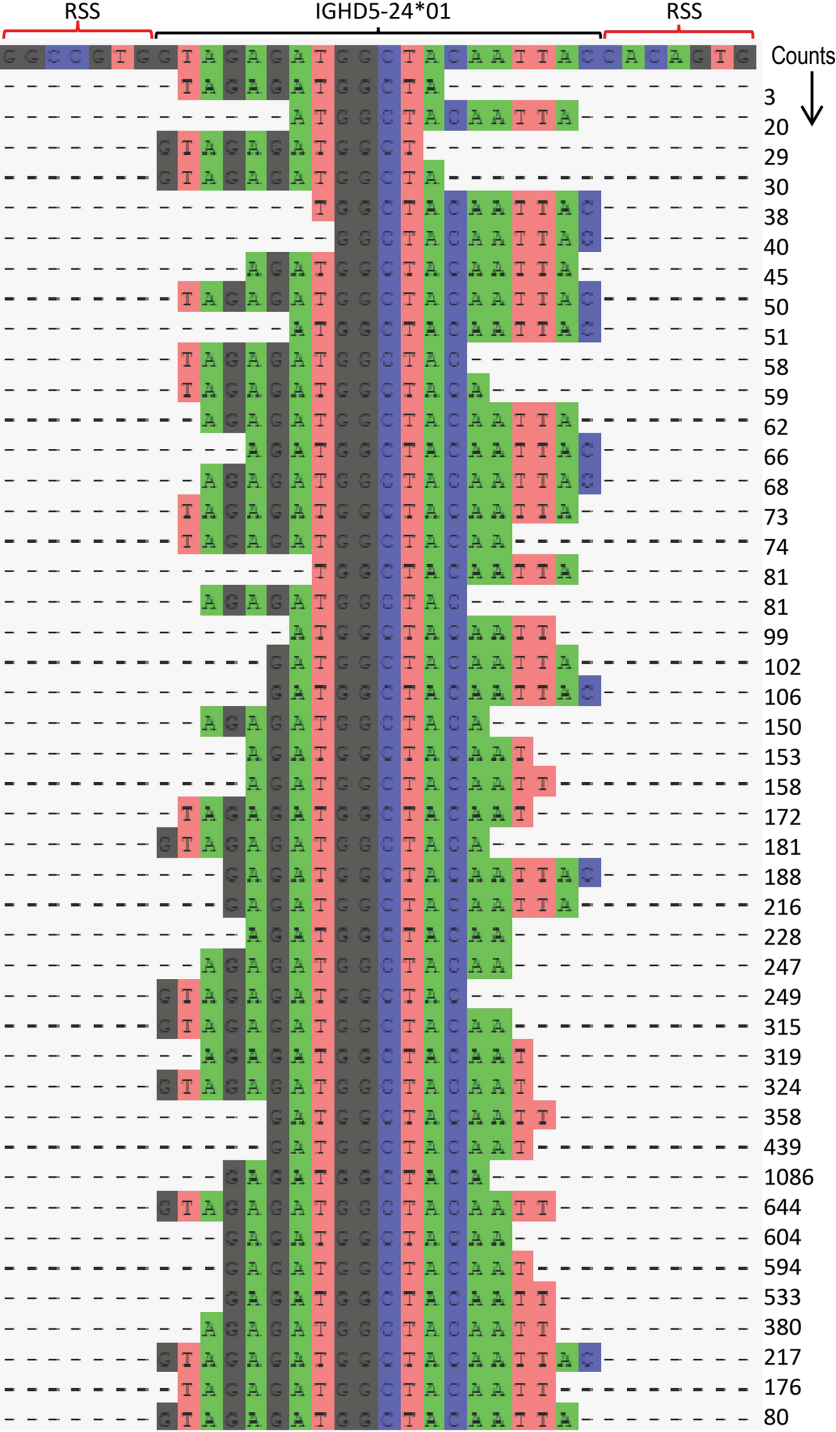
Illustration of IGHD LCS analysis procedure using IGHD5-24*01. The *corecount* LCS procedure identifies allele specific substrings from each sequence present in the supplied IGHD allelic database. These LCS substrings are a minimum percentage of the full length IGHD allele, as defined by the user, in this case 60%. The program searches the MiAIRR formatted library table and produces a set of counts for each of these substrings – with each VDJ sequence analyzed providing a maximum count of 1 of this set. An example is shown for allele IGHD5-24*01 in case D19. In this procedure the program utilizes sequences where the remaining segment of IGHD5-24*01 in the VDJ recombinant is central to the D core, but also enables the use of VDJ recombinations that skew towards the 5’ or 3’ part of the IGHD allele. The position of the V allele and the 5’ and 3’ RSS heptamers are shown in the top row. A total of 45 unique substrings of IGH5-24*01 are shown to be utilized, each resulting in different counts, the sum of which is the corecount total for this IGHD5-24*01 allelic sequence. This approach enables a ***series*** of ‘cores’ to be utilized for D gene analysis rather than restricting it to a single central ‘core’ of that germline.

To meet the special requirements for D genotyping, the *corecount* module contains an additional process designed to maximize IGHD allele counts that utilizes a longest common substring (LCS) process (**Figure 4ii**, right). This takes advantage of the knowledge that the segment of an individual D gene that is incorporated within a VDJ recombination can vary widely between individual recombinations. While the standard *corecount* database truncation process enables accurate IGHD genotyping, it may not provide the maximum counts for IGHD alleles since the amount of sequence loss at the 5’ and 3’ end of D alleles can vary widely during the recombination process. The LCS process takes this into consideration to additionally incorporate the results of recombination associated trimming that are heavily biased to either the 5’ or 3’ end of the D gene. These end-biased D sequences can be non-overlapping with the standard trimmed *corecount* database but can, however, be utilized with an LCS-based process, thereby allowing additional D cores to be utilized in the genotyping of D genes. The module creates a series of substrings of the database IGHD alleles, each of which is necessarily allele specific and encompasses at least 50% the length of the reference D allele. These allele specific substrings are utilized as ‘D-core’ sequences for D gene analysis. In each case, to avoid overcounting, only a single allele specific LCS D-core is counted for a single VDJ sequence. The IGHD allelic counts for each substring of IGHD5-24*01 achieved by the LCS approach is shown for D19 in **Figure 5** and for both D19 and D46 in **Supplemental Table 2**. The *corecount* program registers both individual sequence matches, and individual sequence matches with unique UMIs (**Figure 4iii**).

A critical step in the *corecount* process involves the filtering of the raw counts produced by the program to output a genotype (**Figure 4iv**). This is achieved in several steps. First, the program utilizes an allelic ratio filter that compares allelic sequence counts within each gene. The filtering takes place at the gene level, since heterozygous alleles of the same gene, with a few exceptions, if present in an individual genotype, will be utilized at similar overall frequency. Hence, an allelic ratio of 0.1 will eliminate false positives for V and J genes. Due to the shorter length of D genes a higher allelic ratio of 0.2 is utilized. The program also takes advantage of an expected frequency table filter step that tests the overall frequency of each allele within the unmutated expressed genotype and removes alleles where the count is below a cutoff defined through the analysis of the expression levels of alleles in multiple individuals. In the test case described here the gene specific expression levels were calculated based on the expression of all IGHV genes in the 16-case sample set (**Supplemental Figure 3**). This procedure also enables the *corecount* module to include alleles that are found at lower frequency in the IgM repertoire compared to other alleles of the same gene, for example IGHV1-2*05 compared to the other alleles of IGHV1-2 (*02, *04 or *06). Finally, *corecount* utilizes a filter step that involves examining the diversity of the recombinations specific for that allele. This is done by examining the CDR3 length frequency found for all sequences that match each allelic core sequence.

### Library size constraints

**Figure 6:**
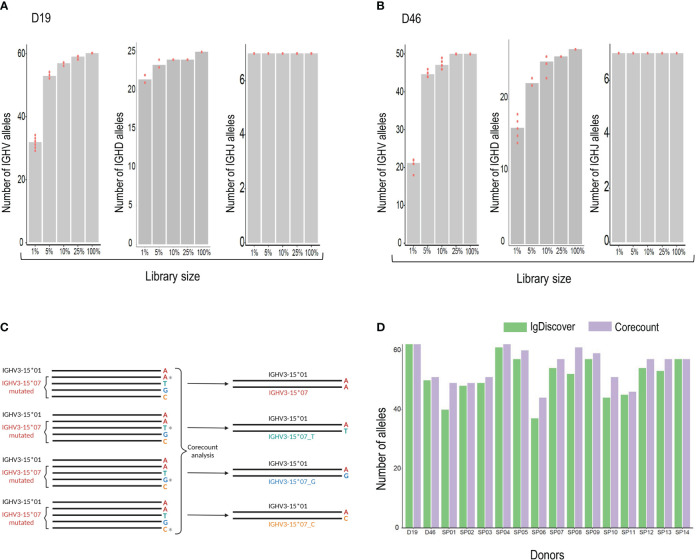
**(A)** Analysis of the effect of library size on genotype output. The number of alleles found by corecount analysis in the complete IgM library (100%) was compared to the numbers found when using libraries that had been reduced to 25%, 10%, 5%, and 1%, each sampled ten times. The Numbers of V, D and J alleles are shown for case D19 and **(B)**, for case D46. **(C)**, corecount identification of allelic end variants. Heterozygous IGHV3-15*07 containing alleles in the D19 IgM library were modified computationally to change the final nucleotide of full length IGHV3-15*07 sequences, an allele that ends in an ‘A’, to alleles that ended on C, G or T nucleotides. The corecount analysis was performed using a database supplemented with the modified IGHV3-15*07 test variants, IGHV3-15*01_C, IGHV3-15*07_G and IGHV3-15*07_T. In each case corecount correctly produced an IGHV genotype containing either the appropriate IGHV3-15*07 sequence (top result) or the modified, IGHV3-15*01_C, IGHV3-15*07_G or IGHV3-15*07_T. **(D)**, Allelic output, based on numbers of alleles identified, from corecount IGHV genotype analysis (purple) was compared to IgDiscover germline inference output (green) for 16 IgM libraries.

Generating a comprehensive expressed genotype from an IgM library is constrained due to differences in the relative frequencies of genes used in productive VDJ recombinations. Some genes, such as IGHV1-18, IGHV3-23, IGHV3-30, and IGHV1-69 are consistently used at high frequencies. Others, such as IGHV1-45, IGHV4-28 and IGHV3-20 are used much less frequently. In the D19 IgM library, for example, there was a 1494-fold difference in frequency between the lowest (IGHV1-45*02) and highest (IGHV1-18*01) allele usage. The result of this frequency difference is that while high frequency genes will be detectable also in IgM libraries of limited size, expression-based analysis designed to produce a comprehensive genotype will inevitably require libraries of sufficient depth to sample the less frequent genes. To test this, we carried out *corecount* analysis of V, D and J alleles using a set of randomly generated samplings of the D19 library. The number of alleles found by *corecount* analysis in the complete D19 IGM library of 925,238 assigned reads was compared to that found in a library of size of 25%, 10%, 5%, 1%, each sampled ten times (**Figures 6A, B** and **Supplemental Table 3**). The results indicated that reduction of the library size to 25% did not impact the identification of most alleles in the genotype, with only the most infrequently utilized genes being missed. Reduction to 10 or 5% resulted in the loss of between 6-10 alleles from each IGHV genotype, while library sizes of 1% of our starting library (corresponding to about 10,000 reads) resulted in a substantial loss of alleles identified with, approximately 25 alleles missing from the *corecount* IGHV genotype of both D19 and D46. IGHJ genotyping was less susceptible to library size constraints with all alleles being identifiable even at the 1% libraries. IGHD, similar to IGHV, shows a loss of allelic detection as library size is reduced with between 5-8 IGHD alleles being lost at the 1% library size (**Figures 6A, B**).

### *Corecount* used to validate alleles containing end variants

A critical issue that impedes both BLAST assignment and inference based genotyping approaches involves the difficulty in assessing alleles where variation occurs in the part of a gene that is subject to high rates of recombination associated change. While *corecount* enables the genotypic identification of variants, such as IGHV1-46*03 or IGHV3-7*04, containing nucleotide differences close to the IGHV 3’ end, the current study shows recombination associated alteration of allelic sequence is particularly heightened in the final two 3’ nucleotides of the V. This area is subject to high level of nucleotide trimming and/or replacement during VDJ recombination; however, the results shown in **Figure 3B** and **Supplemental Figure 1A** indicate that for most alleles in an IgM library, between 20-50% of unmutated V sequences can be identified as full length. This percentage ensures sufficient sequences to enable *corecount* to assess the presence of specific candidate end variant alleles.

To test this, we computationally modified a set of heterozygous allele pairs in the D19 genotype to introduce three additional end variants of one of the heterozygous IGHV allelic pairs. The final nucleotide was modified from A to C, A to G or A to T. **Figure 6C** shows a schematic of the process using the IGHV3-15 heterozygous pair as an example. In this case the IGHV3-15*07 allele was modified to alter the final nucleotide in all library sequences assigned to this allele that were full length. Three modified libraries were produced and analyzed with a *corecount* database supplemented with IGHV3-15*07_C, IGHV3-15*07_G and IGHV3-15*07_T end modified allelic sequences. In each of the three modified library analyses, the appropriate IGHV3-15 genotype was identified by *corecount* (**Supplemental Table 4**). This process was also applied to end variant assessment of the gene IGHV3-48, with *corecount* outputting the appropriate end variant on each occasion (**Supplemental Table 4**).

### Batch genotype analysis using *corecount*

The *corecount* program is designed to genotype both individual libraries and sets of libraries. To test this out we genotyped the 16 cases in our study set using the program for expressed IGHV, IGHD and IGHJ alleles. The results show the expression of a total of 103 unique IGHV alleles, 7 IGHJ alleles and 33 IGHD alleles within the 16 cases (**Supplemental Table 5**). The *corecount* analysis of DNA genotyped case D19 was of particular interest as 3/65 IGHV alleles that were identified during Sanger sequence analysis had not been part of the IgDiscover inferred output. The alleles IGHV2-70*05, IGHV3-30*03 and IGHV3-66*03 did indeed show some counts using the *corecount* analysis program, albeit substantially lower than the counts of other alleles of the respective genes, indicating that in this case, at least, these three alleles are utilized at lower frequency (**Supplemental Table 5**). The *corecount* module enabled a more comprehensive genotype output compared to IgDiscover in 14/16 cases analyzed (**Figure 6D**). In most cases the missed alleles that were genotyped by *corecount* were those of lower frequency genes such as IGHV3-13, IGHV4-28, IGHV2-70, and IGHV3-43 (**Supplementary Table 5**).

## Discussion

The use of AIRR datasets for repertoire analysis is a well-established field of research in many different areas of immunology, vaccination, evolutionary studies and basic biology (20–23). The principle involves the deep sequencing of libraries of a specific subtype, for example IgM, IgG or TRBV, and the assignment of all sequences to a reference database, thereby enabling the identification at the gene or allele level of the recombined sequences in the library (24). The accuracy of the assignment depends on several critical factors. These include the completeness of the reference database, the sequence depth of the library, and the accuracy of the assignment software. The use of germline inference software to produce an individualized germline database for each library is one solution for reference databases that do not reflect the range of alleles in a population (25, 26). However, the inference approach can be computationally expensive in terms of time and the stringency required to avoid false positives means that libraries with lower numbers of sequences may result in an output that misses low frequency alleles. This may be particularly critical in disease studies where a particular association to a gene or allele may be missed if the genotyping is incomplete. As sequence methodologies improve apace, enabling identification of IG and TCR variant germline sequences from multiple human populations, the addition of the newly identified germline sequences to current reference databases will gradually result in well-validated and comprehensive reference sets (17, 27). Inevitably, this will obviate the need for novel germline inference if the resultant database can enable high confidence genotyping. We show how the integration of genomic sequencing and NGS based analysis of Rep-Seq libraries can facilitate the rapid genotyping of V, D and J genes in multiple individuals, with the sequences in the *corecount* IGHV, IGHD and IGHJ databases having been genomically validated to ensure they are full length. We demonstrate how the use of a comprehensive and end-corrected database derived from a subset of individuals can be used to derive to high confidence genotypes in multiple cases.

The availability of an IgM library of high sequence count from an individual whose germline allelic content has been independently validated at the genomic level by a well understood and highly accurate methodology provides an ideal benchmarking dataset, both for the current study and for future computational tool development. Knowledge of the full-length sequences of all IGHV, J and D alleles in case D19 enabled the accurate assessment of the V, D and J regions affected by recombination, both in terms of the frequency of nucleotide deletion or replacement, and the specific parts of the V, D and Js altered during VDJ recombination. While most of the genomic validated alleles confirmed the known allelic sequences found in the commonly utilized IMGT database, we did identify several discrepancies. Allele IGHV5-51*03, for example, in our analysis, appears to be two nucleotides longer than that found in the IMGT database (28), however, in addition to the Sanger validated sequence evidence, two additional factors suggest the 2 nt longer sequence is the correct length of this allele. First, the length of other alleles of this gene, for example, IGHV5-51*01 and IGHV5-51*07, is 296 nts, the same as we find for our Sanger validated sequence. For almost all functional allelic variants of single genes, a consistent length is common, with most length related variations within a single V gene being single codon elongations of framework 1 rather than extensions at the 3’ of the V sequence. Second, within a single library, the counts of unmutated allelic sequences follow a consistent pattern, with the count numbers increasing rapidly as the allelic search string is reduced by single nucleotides on the 3’ end of the V, due to the final two 3’ nucleotides being particular prone to recombination associated truncation. In the case of IGHV5-51*03, the pattern of count increase during single nucleotide deletion is inconsistent with a 294 nt allele length (**Supplemental Figure 1B**). We noticed the same pattern for the extended IGHV3-66*02, IGHV4-38-2*01 and IGHV5-10-1*03 genomically validated alleles. The addition of genomic information is particularly important in the case of IGHV alleles that contain variant nucleotides close to the 3’ end of the gene (29). The 3’ single nucleotide length increase of both IGHJ6*02 and IGHJ6*03 is justified due to the Sanger validated sequences matching the available GRChr37 and GRChr38 assembly sequences, in both cases showing an additional G nucleotide present immediately before the GT splice donor site. In addition, the extension of IGHJ6*02 and IGHJ6*03 results in all functional human IGHJ alleles ending with the 4 bp string ‘TCAG’. The most truncated reference sequence extended with the Sanger validation data was that of IGHV2-70*05, a reference V allele that is truncated by 26 nts at the 5’ end and 38 nts at the 3’ end. Interestingly, despite being present in the genome of D19, the allele was not detectable in the expressed sequence, suggesting that in this case, at least, it was non-functional. The length of additional sequence identified for IGHV2-70*05 is substantial and we cannot rule out that additional extended length alleles may be identified that differ from the sequence identified in this study but also overlap the reference IGHV2-70*05. In this case it may be beneficial that the allele sequence identified here should be assigned a unique allele designation. Importantly, this allelic variant of IGHV2-70 was found only at the genomic level and not in either of the two independent IgM libraries. This is similar to the results we found for the genomically validated IGHV3-30*03 and IGHV3-66*03 alleles in this individual, revealing the advantage of combining expression and genomic based genotypic analysis as a means of defining allelic functionality or even uncovering patterns of variation of usage frequency for particular alleles.

In addition to V genes, the *corecount* module enables the genotyping of J and D genes identified through either genomic sequencing or expression-based inference. We show the presence of variants of IGHD3-10 and IGHD3-16 previously identified using the IgScout program by Safonova et al. (30, 31) as well as a novel variant differing by 1 nt from IGHD5-12*01, identified by IgDiscover (**Supplemental Table 5**). Low frequency alleles such as IGHV7-4-1*01 (32) are also readily genotyped by *corecount*. The development of novel techniques to identify Ig genomic haplotypes (8) confirms the variation seen by inferred haplotype analysis in humans and other species (33–35), revealing a profound heterogeneity within the adaptive immune loci at the individual and population level.

The *corecount* process has a distinct set of advantageous features when applied for the purpose of individual IG genotyping. The process can be easily batched within the program to genotype large numbers of libraries. The user is not required to use the same allele reference set in the production of the original MiAIRR format table that is used for *corecount* analysis, meaning that reanalysis of large numbers of libraries can be quickly performed using updated reference databases. The process can genotype alleles where a variant nucleotide is present at positions particularly refractory to germline inference, such as in the final two 3’ nts of a V sequence. In the case of alleles that show differential frequency of usage, the program can utilize a filtering step that utilizes a table of allele frequencies derived from the analysis of multiple individuals. This process ensures that known low frequency alleles, for example IGHV1-2*05 or IGHV7-4-1*01, are included in the genotype output of cases that contain these alleles. The *corecount* process provides highly complete IG genotypes for size restricted libraries, enabling the inclusion of low frequency alleles in its output. Importantly the *corecount* program is not restricted to IgM libraries. It can also be used for IgK and IgL genotyping, as well as for TRA, TRB, TRG and TRD genotyping (36). It is important to note, however, that since *corecount* utilizes exact matches to expressed sequences, it requires libraries with a substantial proportion of unmutated sequences and so IgG libraries will not be suitable.

Allelic variation within the adaptive immune genes is widespread at both the individual and population level. To date, only limited numbers of genetic polymorphisms within the Ig and TCR loci have been shown to produce clear phenotypic effects (37), or found associated with disease, however the effect of individual differences in germline variant content is increasingly being seen as playing a critical role in providing the necessary immunological diversity to ensure survival of populations following pathogenic exposure.

The careful characterization of IG and TCR polymorphisms at both the genomic and expression level will provide a means to understand the role such variation plays in immune response to pathogens and vaccines (18), and potentially enable the identification of particular genotypes that contribute towards the development of autoimmune conditions. The *corecount* module builds upon the germline inference approach of IgDiscover and can contribute towards the rapid advance in knowledge that is occurring in the field of personalized immunogenetics.

## Materials and methods

### Human samples and ethics statement

PBMCs were isolated using the Ficoll-paqueTM (GE Healthcare Life Sciences) followed by DNA and RNA extraction as described previously (38). RNA was extracted using the RNeasy kit (Qiagen) and genomic DNA was extracted using Gentra Puregene Buccal Cell Kit (Qiagen). PBMCs were extracted from pseudonymized donors, and all genetic data were handled according to local regulations and ethics permits #2016/1053-31 and #2017/852-32, #2020-01620, #2020-02881 and #2020-05630, obtained from Swedish Ethical Review Authority, Stockholm, Sweden.

### IgM library preparation

cDNA was created using the IgM specific primer that contains Illumina’s Read2 sequence and a UMI from 250 ng of extracted RNA as described previously (38). The cDNA was purified using the Qiagen PCR purification spin column kit and resuspended in 20 µl of H2O. Two independent multiplex libraries were created that target 5’ UTR and 5’ leader regions of each functional IGHV gene to enable the production of full-length VDJ amplicons. 5 µl of the purified multiplex product was used as template for index PCR. The indexed libraries were purified using PCR purification kit (Qiagen) followed by magnetic bead purification (AMPure XP magnetic beads, BD) with the purified libraries quantified and sequenced using Illumina version 3 (2 x 300 bp) sequencing kit with 13% of 12 pM PhiX172 DNA as control.

### Computational analysis

#### Germline Inference

IgDiscover inference (IgDiscover version 0.12.4.dev224+g2b41517) was performed as described previously (14, 34) using a starting database derived from the IMGT human reference database, downloaded May 2021. Data from multiple IgDiscover runs was collected using a built-in collect module available within the IgDiscover tool.

#### *Corecount* implementation

*Corecount* is an IgDiscover module that genotypes Rep-Seq libraries that contain a significant proportion of unmutated sequences by counting either 5’ or 3’ trimmed exact occurrences of allele specific query sequences within each library. The process requires an end-corrected allele database and a full length AIRR-seq library, with sequencing read errors similar or lower than produced by the Illumina MiSeq platform. The input for the program is IgBLAST assignments in MiAIRR format and a FASTA file with either V, D or J sequences of interest. The implementation for Vs and Js can be summarized by the following steps: a) check if all input database alleles are unique, b) trim the database sequences using a user specified trim length to create an allele-specific ‘core’ database, c) find exact matches within the MiAIRR format table d) compute statistics such as the maximum CDR3 length and sequence frequency and gene based allelic ratio to perform genotype filtering. Filtering criteria such as CDR3 statistics are used to confirm independent usage of germline sequences, while allelic ratio discards signal from spurious counts due to sequencing errors. Additionally, an excepted gene frequency table can be provided to exclude alleles that appear due to index errors and to include alleles that are expressed at lower frequency than other alleles of that specific gene.

#### Genomic validation

Genomic DNA from non-B cells was isolated using the Gentra Puregene Buccal Cell Kit (Qiagen). 10 ng of the isolated DNA was amplified using gene-specific primers. Primers were designed using the GRChr37 and GRChr38 human assembly reference genomic sequences. For V genes the forward primer was upstream of the L1 exon coding sequence and the reverse primer was downstream of the L2 exon RSS sequence. For both D and J genes the primers were designed to amplify a segment that completely encompasses both 5’ and 3’ RSS sequences for the D genes and the 5’ RSS and the downstream splice site for the J genes. Primers used in genomic validation are shown in **Supplemental Table 6**. Amplification conditions were denaturing at 95°C for 20 seconds, annealing at 64°C for 30 seconds and elongation at 72°C for 40 seconds using the KAPA HiFi Hotstart readymix system (Kapa Biosystems). The PCR product was gel purified using Gel purification kit (Qiagen) and ligated into CloneJET pJET 1.2 vector (Thermo scientific) and XL10 gold ultracompetent *E.Coli* cells (Agilent technologies) were used for transformation with 1-2 µl of ligation mixture. The transformed cells were allowed grow overnight at 37°C on 100 mg/ml LB agarose plate with ampicillin. Colonies were picked and screened allowing the positive colonies to be grown overnight in a shaking incubator at 220 rpm, 37°C in LB medium with ampicillin. Plasmid was isolated using GeneJET plasmid miniprep kit (Thermo scientific) and were Sanger sequenced using T7 forward primer (https://www.genewiz.com/en-GB/Public/Services/Sanger-Sequencing) and the pJET1.2 reverse primer.

### Genotype analysis

The *corecount* module is a python-based process that utilizes the filtered sequences from IgDiscover, present in the filtered.tsv.gz file in the iteration 1 results folder. The *corecount* module requires an end-corrected reference database containing an allele set that encompasses all the alleles present in the test library and contains several user defined parameters to enable genotype analysis of IG and TCR libraries. The *corecount* module looks for 100% match to allele specific sequences, the allelic ‘core’. These are allelic sequences that are computationally truncated to remove a set number of nucleotides in the immediate vicinity of the recombination region, thereby maximizing the count of sequences for each allele. For each gene, the number of sequences identical to specific ‘core’ alleles in the database are determined and processed using a set of user adjustable filters to ensure genotypic accuracy. These filters are: allelic ratio, based on the expected maximum ratio of counts of alleles of the same gene (default setting 0.1); minimum acceptable count; and LENMAXFREQ, a filter based on CDR3 length diversity. The *corecount* analysis was performed on V, D and J sequences from both the libraries.

### Haplotype analysis

The IgDiscover contains a module known as *plotalleles* for generating inferred haplotypes. This module works on the principle that heterozygous J or D alleles can be used to assign V alleles to their appropriate haplotype due to VDJ recombination occurring locally on a single chromosome. Therefore, for genes from the maternally derived haplotype, V alleles will associate with only maternally derived J or D alleles and likewise for genes on the paternal haplotype V alleles will recombine with downstream J or D alleles from the paternal derived haplotype. This process creates a map of V alleles, the order of which is user defined (in this case it is the known order of V genes found in the GRChr38 genomic assembly).

### End variant modification analysis

Heterozygous alleles of IGHV3-15 and IGHV3-48 from the D19 library were modified using a custom python script by altering nucleotides at specific positions within a V sequence. In the case of final nucleotide alteration tests the script ensured that only sequences for the allele to be modified were untruncated within the VDJ sequence. As a result, three alternative libraries were produced for each single nucleotide modification and the four full length allelic test variants (original plus the three single nt variants) were included in the *corecount* database. In this scenario *corecount* finds four alleles of the same gene in the database, all of which show variation only at the final nucleotide, resulting in the program not reducing the length of the core sequence but instead using the full length of all 4 ‘alleles’ of this gene, during *corecount* genotyping.

### Additional software

The dendrograms were made using Aliview (version 1.26) and fasttree (version 2.1.11). Plots were made using R packages ggplot (version 3.1.0), gplots (version 3.0.1.1) and dplyr (version 0.8.0.1).

## Data availability statement

The data produced in the study were deposited in accordance with the GDPR guidelines at the SciLifeLab Data Center, the digital object identifier 10.17044/scilifelab.21618150. The sanger validated alleles in this study from donor D19 have been deposited at the Genbank repository, with the accession numbers: J alleles (ON023001-ON023007), D alleles (ON052093 - ON052119) and V alleles (ON052029 - ON05209 and OP913455). The corecount program running parameters for IGHV, IGHD, and IGHJ genotype analysis, in addition to the IGHD trimming table, expected frequency table for standard IGHV allelic genotyping and gene-based genotyping are provided in the corecount GitLab repository at the following link: https://gitlab.com/gkhlab/corecount-genotyping.

## Ethics statement

The studies involving human participants were reviewed and approved by Swedish Ethical Review Authority, Stockholm, Sweden. The patients/participants provided their written informed consent to participate in this study.

## Author contributions

Conceptualization, MMC and GBKH. Methodology, SN, MK, MC, and MMC. Investigation, SN, MK, MC, and MMC. Visualization, SN and MMC. Funding acquisition, GBKH. Supervision, GBKH and MMC. Writing – original draft, MMC, SN, and GBKH. Editing – original draft, all authors. All authors contributed to the article and approved the submitted version.

## Supplementary material

Supplementary Figure 1 Analysis of IGHV gene 3’ end truncation. **(A)** Effect of recombination based 3’ nucleotide alteration on unmutated allele counts in case D46. The proportion of counts for V alleles containing the full-length V sequence shown in blue. **(B)** Effect of truncation of allele sequence string on counts of unmutated allele sequences in case D19. The proportion of counts of a series of single nucleotide 3’ deletions are shown compared to the counts of a 6 nt truncation at the 3’ end of the V sequence. The proportion of full-length sequences for each allele are shown on the left side of each barchart with the corresponding proportion of 3’ truncated sequences -1 to -5 shown for each allele for the IGHV2 to IGHV7 family genes. **(C)** Effect of truncation of allele sequence length on the proportion of counts for the heterozygous IGHV1-46 and IGHV3-7 genes, both of which show 3’ end variation, in case D46.

Supplementary Figure 2 Analysis of IGHJ and IGHD VDJ associated truncation. **(A)** Effect of truncation of IGHJ sequence string on the proportion of IGHJ allele counts in case D19. The proportion of full-length sequence counts for each J allele are shown on the left side of each barchart with the corresponding proportion of 5’ truncated sequences -1 to -9 shown **(B)** Effect of truncation of IGHD allelic sequence string on the proportion of IGHD allele counts in D19. The proportion of full-length sequence counts for each D allele are shown on the left side of each barchart with the corresponding proportion of dual direction truncated sequences -1 to -4 shown.

Supplementary Figure 3 Analysis of unmutated gene frequency in the 16-case sample set. The frequency of unmutated sequences from alleles of each expressed IGHV gene was calculated based on the frequency of unique UMI counts within the full unmutated repertoire. These are represented as box plots per gene.

Supplementary Table 1 Sanger validated IGHV, IGHD and IGHJ alleles from case D19.

Supplementary Table 2 LCS IGHD genotype analysis and allelic ratio cutoff test.

Supplementary Table 3 Library size analysis.

Supplementary Table 4 End variant analysis.

Supplementary Table 5 Genotypes. 1. Final genotypes IGHV 16 cases 2. Final genotypes IGHD 16 cases 3. Final genotypes IGHJ 16 cases 4. D19_unfiltered_IMGT_DB 5. D19_zygosity 6. Common_gene_corecount_6_cases 7. D19_individualized_no_filter 8. Allele_only_in_corecount

Supplementary Table 6 Validation Primers.
