# Supplementary figures and images for: Adaptive immune receptor genotyping using the corecount program
Source: Front Immunol. 2023 Apr 11;14:1125884. doi: 10.3389/fimmu.2023.1125884 (PMC10126697; doi:10.3389/fimmu.2023.1125884)

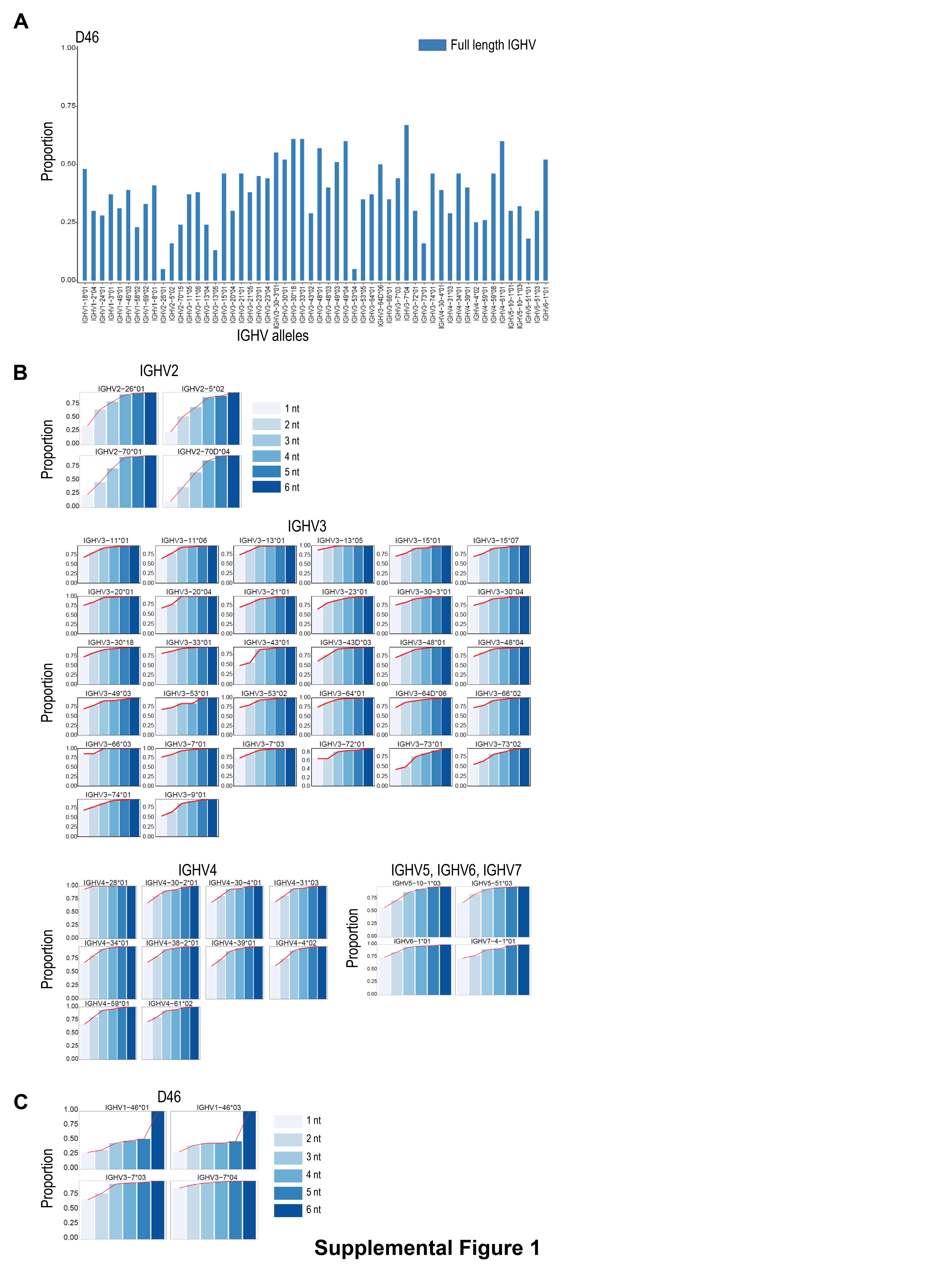

Supplement: Supplementary Figure 1 — Analysis of IGHV gene 3’ end truncation. (A) Effect of recombination based 3’ nucleotide alteration on unmutated allele counts in case D46. The proportion of counts for V alleles containing the full-length V sequence shown in blue. (B) Effect of truncation of allele sequence string on counts of unmutated allele sequences in case D19. The proportion of counts of a series of single nucleotide 3’ deletions are shown compared to the counts of a 6 nt truncation at the 3’ end of the V sequence. The proportion of full-length sequences for each allele are shown on the left side of each barchart with the corresponding proportion of 3’ truncated sequences -1 to -5 shown for each allele for the IGHV2 to IGHV7 family genes. (C) Effect of truncation of allele sequence length on the proportion of counts for the heterozygous IGHV1-46 and IGHV3-7 genes, both of which show 3’ end variation, in case D46. [file Image_1.jpeg]

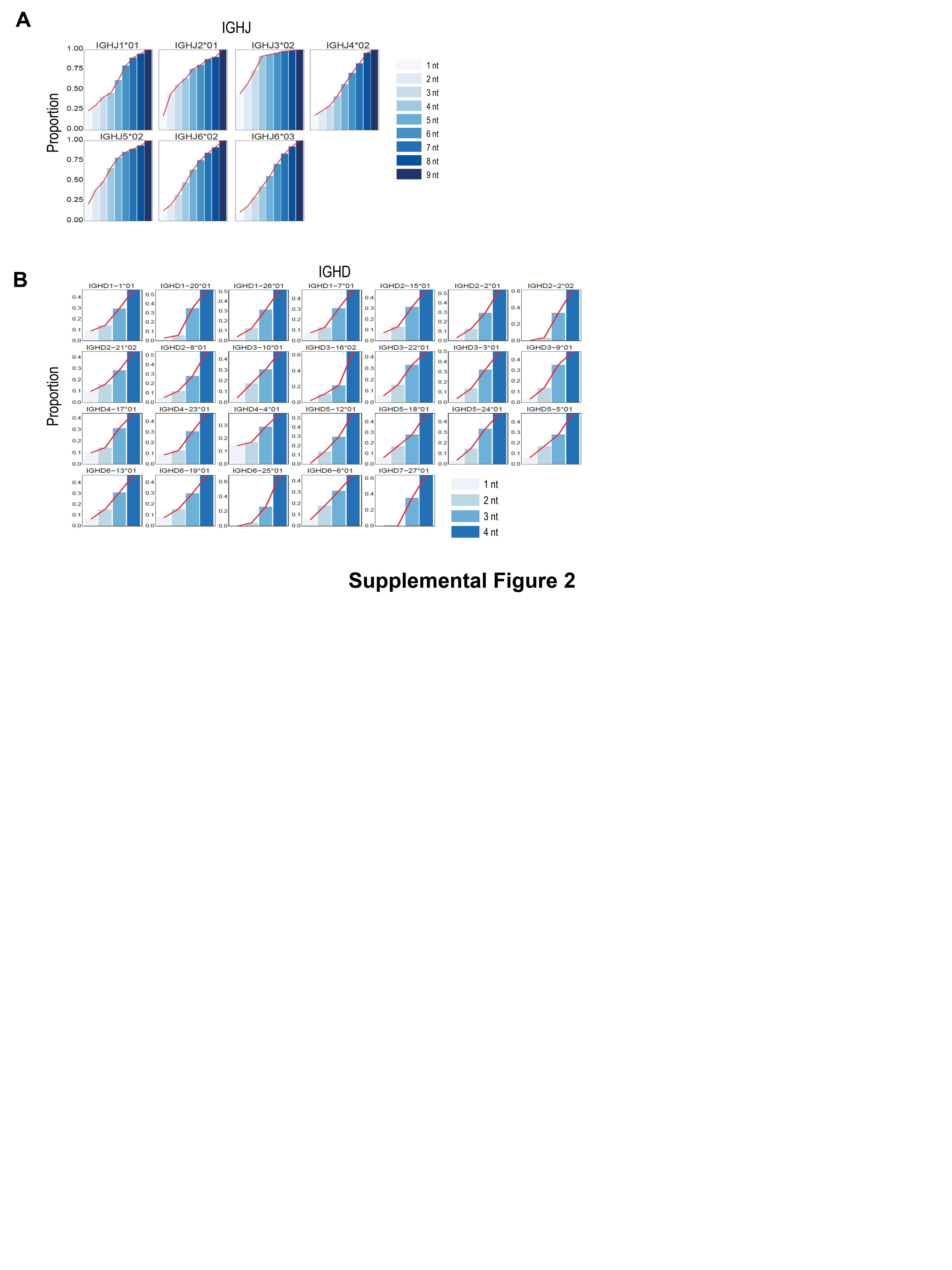

Supplement: Supplementary Figure 2 — Analysis of IGHJ and IGHD VDJ associated truncation. (A) Effect of truncation of IGHJ sequence string on the proportion of IGHJ allele counts in case D19. The proportion of full-length sequence counts for each J allele are shown on the left side of each barchart with the corresponding proportion of 5’ truncated sequences -1 to -9 shown (B) Effect of truncation of IGHD allelic sequence string on the proportion of IGHD allele counts in D19. The proportion of full-length sequence counts for each D allele are shown on the left side of each barchart with the corresponding proportion of dual direction truncated sequences -1 to -4 shown. [file Image_2.jpeg]

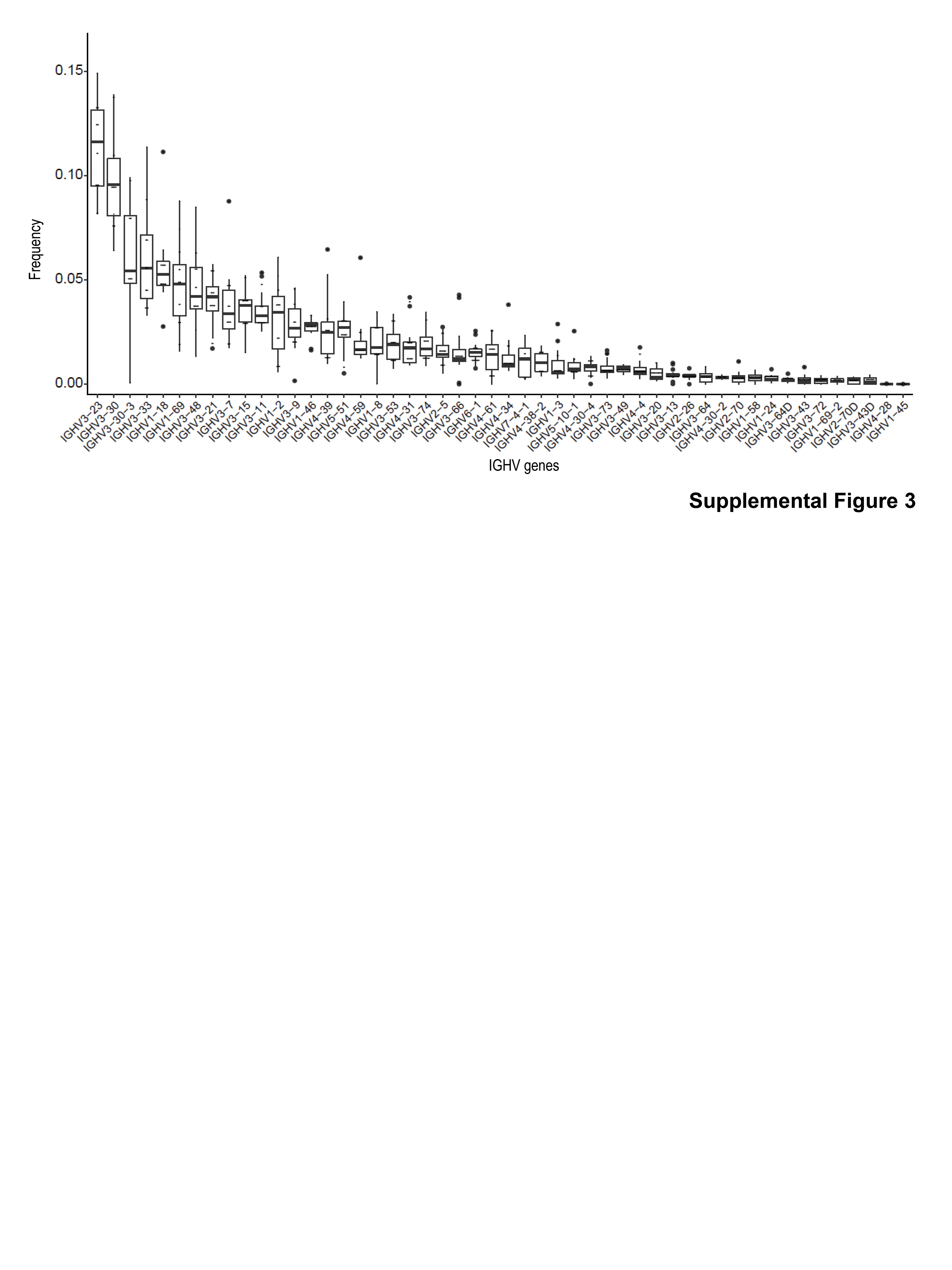

Supplement: Supplementary Figure 3 — Analysis of unmutated gene frequency in the 16-case sample set. The frequency of unmutated sequences from alleles of each expressed IGHV gene was calculated based on the frequency of unique UMI counts within the full unmutated repertoire. These are represented as box plots per gene. [file Image_3.jpeg]
